# Implementation of case-based learning plus flipped classroom in standardized residency training for ultrasound physicians

**DOI:** 10.12669/pjms.42.2.13022

**Published:** 2026-02

**Authors:** Weina Mu, Xin Liu, Hongwei Chen, Ying Liu, Juan Liu, Wei Liu

**Affiliations:** 1 Weina Mu Department of Ultrasound, Baoding NO.1 Central Hospital, Baoding, 071000, Hebei, China. Baoding Key Laboratory of Echocardiography, Baoding, 071000, Hebei, China; 2 Xin Liu Department of Ultrasound, Baoding NO.1 Central Hospital, Baoding, 071000, Hebei, China. Baoding Key Laboratory of Echocardiography, Baoding, 071000, Hebei, China; 3 Hongwei Chen Department of Ultrasound, Baoding NO.1 Central Hospital, Baoding, 071000, Hebei, China. Baoding Key Laboratory of Echocardiography, Baoding, 071000, Hebei, China; 4 Ying Liu Department of Ultrasound, Baoding NO.1 Central Hospital, Baoding, 071000, Hebei, China. Baoding Key Laboratory of Echocardiography, Baoding, 071000, Hebei, China; 5 Juan Liu Department of Ultrasound, Baoding NO.1 Central Hospital, Baoding, 071000, Hebei, China. Baoding Key Laboratory of Echocardiography, Baoding, 071000, Hebei, China; 6 Wei Liu Department of Ultrasound, Baoding NO.1 Central Hospital, Baoding, 071000, Hebei, China. Baoding Key Laboratory of Echocardiography, Baoding, 071000, Hebei, China

**Keywords:** Case-based learning, Flipped classroom, Residency, Standardized training, Teaching method

## Abstract

**Objective:**

To evaluate the effectiveness of integrating case-based learning (CBL) with the flipped classroom approach in the standardized residency training of ultrasound physicians.

**Methodology:**

This was a retrospective study. A total of 26 residents undergoing standardized ultrasound residency training at Baoding NO.1 Central Hospital between January to August 2024 were enrolled in this study. Participants were randomly assigned to an experimental group (*n=* 13) and a control group (*n=* 13). The experimental group received CBL and flipped classroom instruction, while the control group was taught using traditional methods. Theoretical examination scores, perceived learning outcomes and teaching satisfaction were compared between the two groups.

**Results:**

The experimental group achieved significantly higher theoretical examination scores than the control group (*p<* 0.05). Participants in the experimental group also demonstrated significantly improved learning outcomes and reported higher satisfaction with the teaching methods compared with those in the control group (*p<* 0.05, respectively).

**Conclusion:**

The teaching approach combining CBL and the flipped classroom model may enhance clinical reasoning, stimulate learning motivation, foster innovation and contribute to the overall effectiveness of standardized ultrasound residency training.

## INTRODUCTION

Standardized residency training plays a critical role in cultivating future medical professionals, emphasizing competency-based education to improve both theoretical knowledge and practical skills to the greatest extent.^1^ Unlike traditional undergraduate medical education, residency training demands not only a solid foundation of medical knowledge but also a high level of clinical competence.^2^ A major challenge in residency education is transforming abstract theoretical content into engaging, memorable and clinically relevant learning experiences that can stimulate learners’ enthusiasm and optimize educational outcomes.^3^

Case-based learning (CBL) is a pedagogical strategy centered on real or simulated clinical cases.^4^-^7^ It places learners in realistic clinical scenarios, encouraging active participation and critical thinking. In this model, instructors act as facilitators, guiding learners through the process of analyzing complex cases and making reflective judgments. The flipped classroom is another student-centered instructional approach,^8^-^10^ wherein learners independently acquire foundational knowledge before class through textbooks, videos, or research articles. In-class time is used for interactive learning activities, such as presentations, discussions and group problem-solving under the guidance of an instructor. When combined, the CBL and flipped classroom approaches shift the focus from passive reception in traditional teacher-centered instruction settings to active construction of knowledge, helping learners deepen their understanding and foster long-term knowledge retention. This study explored the application of this hybrid model in ultrasound residency training and evaluates its utility.

## METHODOLOGY

This was a retrospective study. A total of 26 residents undergoing standardized training in the Ultrasound Department of Baoding NO.1 Central Hospital between January to August 2024 were enrolled in this study, randomly divided into an experimental group (*n=* 13) and a control group (*n=* 13). For sampling, a notice of voluntary participation in the study was issued by the head nurse. The experimental group (*n =* 13), comprising two males and 11 females, with a mean age of 26.00 ± 2.12 years; and a control group (*n =* 13), also comprising two males and 11 females, with a mean age of 26.15 ± 2.97 years. In terms of educational background, the experimental group included 7 undergraduates and 6 postgraduates, while the control group included 6 undergraduates and 7 postgraduates. The experimental group received instruction through a combination of CBL and flipped classroom methodologies, whereas the control group was taught using traditional teaching methods. No significant differences were observed between the two groups in sex, age, or educational background (all *p>* 0.05).

### Ethical Approval:

The study was approved by the Institutional Ethics Committee of Baoding NO.1 Central Hospital (No.: [2023]126; Dated: November 27, 2023) and written informed consent was obtained from all participants.

### Teaching Methods:

Control Group: Teaching was conducted according to the standard curriculum using conventional instructional methods focusing on in-class questioning and after-class assignments. Instruction covered typical and atypical ultrasound imaging features of a range of diseases, along with relevant laboratory tests, radiologic examinations and differential diagnoses. Clinical reasoning and key concepts were summarized.

### Experimental Group:

According to the curriculum, the supervising instructor announced the case topic and related content one week prior to each session. One resident was designated to lead the session and was responsible for independently preparing in advance. Preparatory work included reviewing the ultrasound characteristics of the disease, relevant laboratory and imaging findings and differential diagnoses. Learners were encouraged to consult textbooks, literature and online medical resources, with guidance provided by the instructor in gathering and analyzing case-related data. During the classroom session, the designated resident led the presentation, posed questions and facilitated group discussion. The instructor provided feedback, supplemented key learning points and introduced relevant advances and emerging technologies to ensure a comprehensive understanding of the case.

### Outcome Evaluation:

Theoretical Assessment: The theoretical examination consisted of two parts: objective multiple-choice questions covering fundamental knowledge (60 points) and case analysis questions (40 points), with a total possible score of one hundred. The assessment was conducted in a closed-book format. All questions and grading were independently prepared and scored by an associate chief physician with extensive teaching experience to ensure objectivity and consistency.

### Learning Effectiveness Questionnaire:

An anonymous questionnaire was administered to both groups to evaluate self-reported learning gains. The survey assessed improvements in the following domains: clinical reasoning ability, information retrieval skills, anatomical plane recognition, report writing skills, verbal communication, teamwork and collaboration and professional development. Responses were categorized as either “Improved” or “Not Improved.”

### Teaching Satisfaction Questionnaire:

An anonymous satisfaction survey was conducted to assess learners’ perceptions of the teaching performance of instructors throughout the training sessions. Satisfaction was rated as either “Satisfied” or “Not Satisfied.”

### Statistical Analysis:

All statistical analyses were performed using SPSS 21.0. Categorical variables were expressed as frequencies and percentages (*n*[%]) and analyzed using the chi-square (χ^2^) test. Continuous variables were expressed as mean ± standard deviation (*x̄*±*s*) and analyzed using the independent-samples t-test. A *P*-value of less than 0.05 was considered statistically significant.

## RESULTS

Residents in the experimental group achieved significantly higher scores in both the objective and case analysis sections of the written examination compared with the control group (*p<* 0.05). Table-I.

**Table-I:** Comparison of theoretical assessment scores (*χ̅*±*S*).

Group	n	Theoretical Assessment Score		
		Multiple-choice Questions	Case Analysis	Total Score
Experimental	13	50.15±4.04	33.46±2.07	83.62±4.79
Control	13	45.46±6.40	28.77±3.49	75.54±6.28
*t-value*	—	2.236	4.170	3.688
*p-value*	—	0.035	<0.001	0.001

The proportion of residents in the experimental group reporting improvements in clinical reasoning, information retrieval, anatomical plane recognition, report writing, verbal communication, teamwork and collaboration and professional development was significantly higher than in the control group (all *p<* 0.05) Table-II.

**Table-II:** Comparison of self-reported learning outcomes (n[%]).

Group	n	Clinical reasoning	Information retrieval	Anatomical plane recognition	Report writing	Verbal communication	Teamwork and collaboration	Professional development
Experimental	13	13 (100%)	12 (92.37%)	12 (92.37%)	13 (100%)	13 (100%)	12 (92.37%)	13 (100%)
Control	13	9 (69.23%)	7 (53.85%)	6 (46.15%)	8 (61.54%)	9 (69.23%)	6 (46.15%)	8 (61.54%)
*χ^2^*	—	4.727	4.887	6.500	6.190	4.727	6.500	6.190
*p-value*	—	0.030	0.027	0.011	0.013	0.030	0.011	0.013

The experimental group reported significantly higher satisfaction with the teaching process compared with the control group (*p<* 0.05) Table-III.

**Table-III:** Comparison of teaching satisfaction between groups (n[%])

*Group*	*n*	*Not Satisfied*	*Satisfied*
Experimental	13	0(0%)	13(100%)
Control	13	4(30.77%)	9(69.23%)
*χ^2^*	—	—	4.727
*P-value*	—	—	0.030

## DISCUSSION

Through the application of different teaching methods, this study found that residents in the experimental group, who were exposed to the combined CBL and flipped classroom approach, achieved significantly higher theoretical examination scores compared with those in the control group (*p<* 0.05). Moreover, participants in the experimental group showed significant improvements in clinical reasoning, literature retrieval, anatomical plane recognition, report writing, verbal communication, teamwork and collaboration and professional development (*p<* 0.05, respectively). The satisfaction survey also revealed a strong learner preference for the hybrid approach, with significantly higher satisfaction rates in the experimental group than in the control group (*p<* 0.05). This approach also fostered more positive and collaborative instructor–learner relationships through enhanced interaction. These findings are consistent with previous studies,^11^ which have shown that diversified teaching models effectively stimulate learner motivation, enhance the development of clinical reasoning and improve overall teaching effectiveness. However, such integrated instructional strategies place higher demands on clinical instructors, requiring not only comprehensive professional knowledge and technical expertise but also the ability to provide timely, constructive guidance. High-quality teaching is essential to achieving the goals of standardized residency training and improving educational satisfaction.^12^,^13^

Ultrasound medicine is a highly specialized and practice-intensive clinical discipline with wide-ranging applications across various medical specialties.^14^,^15^ It occupies a unique and increasingly important role in diagnostic imaging.^16^ In China, standardized residency training serves as a critical component of postgraduate medical education, emphasizing the development of clinical reasoning and practical skills.^17^,^18^ The primary goal of ultrasound residency training is to cultivate physicians with strong professional ethics, comprehensive clinical competence, effective patient management skills, communication and teamwork abilities and the capacity for continuous learning and teaching for independent, standardized diagnosis and treatment of common and frequently-occurring diseases in clinical practice.^19^ A solid theoretical foundation is essential for mastering operational techniques and ensuring accurate diagnostic interpretation. Traditional teaching methods in ultrasound education are typically instructor-centered, characterized by unidirectional information delivery. Learners often struggle to fully comprehend the abstract and complex theoretical content of ultrasound and the monotonous nature of lectures combined with rote memorization limits their ability to integrate theory with clinical practice. Although conventional instruction may include some in-class questioning, meaningful interaction between instructors and learners remains limited, creating a perceived gap that can hinder engagement and knowledge retention.

In contrast, the combined use of CBL and the flipped classroom model represents a departure from traditional pedagogical approaches. This hybrid method positions the learner at the center of the educational process, with instruction anchored around clinical cases and guided by teaching faculty. Learners are encouraged to actively acquire knowledge by engaging in self-directed learning (*e.g*., reviewing the literature, accessing diagnostic imaging through the PACS system, studying relevant ultrasonographic features) while also honing their communication skills through simulated patient interactions. This transition from passive reception to active inquiry fosters intrinsic motivation and cultivates a proactive learning attitude.

During classroom sessions, learners present clinical cases, lead discussions and engage in critical analysis. These activities enhance classroom interactivity and make learning more dynamic and enjoyable. Teaching faculty offer timely feedback and guidance, facilitating a collaborative learning environment. In this hybrid model, the instructor functions like a conductor of an orchestra, setting the learning framework, pace and direction, while learners acquire knowledge through discussion, cooperation and shared inquiry. The process of case preparation involves summarizing medical histories, identifying and describing key ultrasonographic features, developing diagnostic and differential diagnostic rationales and exploring related clinical knowledge. These tasks require extensive information gathering. Classroom presentations and group discussions further strengthen clinical reasoning and verbal communication skills while promoting teamwork and collaboration among peers.

Beyond knowledge transmission, residency education must also focus on teaching residents how to learn and generate learning motivation. As the saying goes, “*Give a man a fish and you feed him for a day. Teach a man to fish and you feed him for a lifetime*.” Effective pedagogical approaches should aim to cultivate lifelong learning skills and prepare residents to meet the demands of future clinical practice, thereby producing competent and innovative medical professionals.^20^

### Limitations:

However, a small number of samples is the limitation of the study. In view of this, Future studies will incorporate multicenter data and advanced teaching method. The conclusions drawn here require further validation through prospective, large-scale, randomized controlled, and multicenter studies.

## CONCLUSIONS

The implementation of a combined CBL and flipped classroom approach may markedly enhance residents’ clinical reasoning abilities, stimulate enthusiasm for learning and foster innovation. This hybrid teaching model may offer a valuable strategy for advancing the quality and effectiveness of standardized ultrasound residency training programs.

### Authors’ Contributions:

**WM** and **XL:** Carried out the studies, drafted the manuscript and are responsible and accountable for the accuracy or integrity of the work. **HC** and **YL:** Performed the statistical analysis and participated in its design. **JL** and **WL:** Collected the data, performed the analysis, critical review, were involved in the writing of the manuscript. All authors read and approved the final manuscript.
